# Bangladeshi Case Series of Bardet–Biedl Syndrome

**DOI:** 10.1155/2023/4017010

**Published:** 2023-04-15

**Authors:** Fariah Osman, Md Iftekher Iqbal, M. Nazrul Islam, Syed Jahangir Kabir

**Affiliations:** ^1^Ispahani Islamia Eye Institute and Hospital, Dhaka, Bangladesh; ^2^Glaucoma Department, Ispahani Islamia Eye Institute and Hospital, Dhaka, Bangladesh; ^3^Department of Ophthalmology, Bangladesh Institute of Research and Rehabilitation for Diabetes Endocrine and Metabolic Disorders (BIRDEM), Dhaka, Bangladesh

## Abstract

A rare multisystemic, ciliopathic autosomal recessive disorder called Bardet–Biedl syndrome (BBS) primarily affects children of consanguineous marriages. Both men and women are affected by it. It is characterized by some major and many minor features to aid in the clinical diagnosis and management. Here, we reported two Bangladeshi patients (a 9-year-old girl and 24-year-old male) who were presented with various major and minor features of BBS. Both patients came to us with the symptoms including excessive weight gain, poor vision, and learning disabilities with polydactyly. Our case 1 presented four primary features (retinal degenerations, polydactyly, obesity, and learning deficits) and six secondary features (behavioral abnormality, delayed development, diabetes mellitus, diabetes insipidus, brachydactyly, and LVH), whereas case 2 presented five major criteria (truncal obesity, polydactyly, retinal dystrophy, learning disabilities, and hypogonadism) and six minor criteria (strabismus and cataract, delay in speech, behavioral disorder, developmental delay, brachydactyly and syndactyly, and impaired glucose tolerance test). We diagnosed the cases as BBS. Because there is no specific treatment for BBS, we highlighted the importance of diagnosing it as early as possible so that comprehensive and multidisciplinary care can be offered to prevent avoidable morbidity and mortality.

## 1. Introduction

Bardet–Biedl syndrome (BBS) is a rare genetic multisystem disease caused by primary cilia malfunction. Rod-cone dystrophy, kidney abnormalities, postaxial polydactyly, learning impairments, central obesity, and hypogonadism distinguish this ciliopathy. So far, twenty-one BBS-causing genes have been identified (BBS1-BBS21) [1].

First, Bardet in 1920 and then Biedl in 1922 described this syndrome [2]. It mainly occurs in children born from consanguineous marriages. More than half of the studies show that women are affected more frequently than males. Moreover, functional and morphological abnormalities are observed in up to 90% of the affected patients [3].

Its prevalence in Europe and North America is 1 : 100,000. It also occurs more frequently in the Bedouin population of Kuwait, affecting about 1 in 13,500 newborns [4]. In Bangladesh, neither the incidence nor the prevalence of BBS is well-known, and so does from the other Asian countries.

Many resource-constrained hospitals in developing countries, such as Bangladesh, lack genetic testing for BBS confirmation. As a result, BBS is primarily diagnosed based on clinical features and family history. These individuals often exhibit symptoms within the first ten years of life, with impaired night vision being the most common due to retinal degeneration [1]. Although some manifestations, such as polydactyly or genitourinary anomalies, can be detected during pregnancy, such a diagnosis is hardly ever established in early infancy without a positive family history and confirmed molecular testing [5].

The presence of at least four primary features or three primary features and at least two secondary features, according to Beales et al. diagnostic criteria, is required for the diagnosis of BBS (Table 1) [6].

Obesity, which affects 72-92% of BBS patients, appears throughout the first three years of life. The birth weight is typically average, and weight gain begins around the first year [1, 5].

A thorough review of the literature showed no documented case series from Bangladesh. This case series is aimed at increasing understanding of this unique genetic condition among the region's physicians, pediatricians, and ophthalmologists, as the syndrome's rarity and slowly progressing course make early diagnosis challenging. Late detection can lead to increased mortality and morbidity.

## 2. Clinical Presentation

### 2.1. Case 1

A 9-year-old Bangladeshi girl was brought by her parents with complaints of the painless, gradual dimness of vision, especially at night, in both eyes from around six years of her age. They also informed us about her becoming obese, not having average mental growth, inability to concentrate on reading or learning anything, and having three-times convulsions at the age of one year. They also complained of polyuria, polydipsia, and weight loss for the previous three months.

She was the third issue of her non-consanguineous parents, delivered at full-term vaginally without complications.

Her two older siblings led regular, healthy lives and so did the other family members. She was behind her siblings in developmental milestones, such as walking and speaking. She also suffered from a learning impairment and never went to school.

On general examination, she looked apathetic; her vital signs were within normal limit, weight: 34 kg (between 75^th^ and 90^th^ percentile), height: 119.2 cm (below 3^rd^ percentile), obese (BMI: 23.94 kg/m^2^; >95^th^ percentile), and below average intelligence.

Psychiatric evaluation showed severe cognitive impairment which was evident in reduced perceptual reasoning, attention ability, and functional independence.

**Figure 1:**
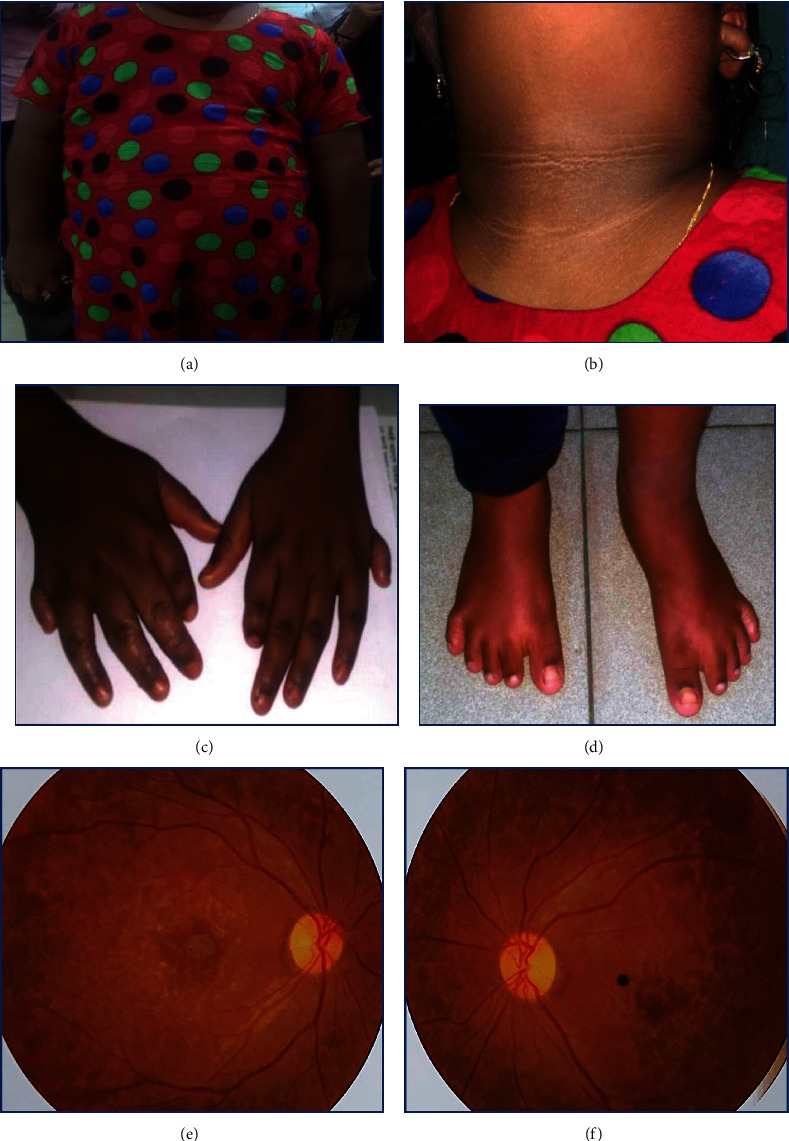
(a) Truncal obesity. (b) Acanthosis nigricans on the neck. (c) Bilateral upper limbs postaxial polydactyly. (d) Short, broad, and stubby fingers on the lower limbs. (e, f) Fundoscopic pictures of both eyes showing “waxy” optic disc pallor and pigmentary retinopathy.

Further examination revealed truncal obesity (Figure 1(a)), no organomegaly or other abnormalities, and acanthosis nigricans on the neck (Figure 1(b)).

Examination of the extremities revealed polydactyly in both hands (Figure 1(c)) and the lower limbs' short, broad, and stubby fingers (Figure 1(d)).

On ocular examination, we could not assess her visual acuity, color vision, or field of vision. Ocular adnexa and anterior segment examination revealed no abnormality.

On dilated fundus examination, both eyes showed pale optic discs, mild arteriolar narrowing, and scattered yellow-white spots with retinal pigmentary changes (most numerous near the equator) (Figures 1(e) and 1(f)).

Investigations were within normal range, including a complete blood count, urine analysis, renal function tests, and chest X-ray. Electrocardiography (ECG) showed features of LVH, which was confirmed by echocardiography. The hearing assessment was normal. Her random blood glucose was 205 mg/dL (normal: 200 mg/dL or above, diabetes), HbA1C 7.2% (normal: <5.5%), and hormone analysis revealed serum TSH 15.02 pmol/L (normal: 10.09 *μ*IU/mL, hypothyroidism). Fasting serum lipid profile showed 269 mg/dL (normal: <150 mg/dL) triglyceride (TG), 192 mg/dL (normal: <200 mg/dL) cholesterol, 41 mg/dL (normal: >35 mg/dL) high-density lipoprotein (HDL), and 127 mg/dL low-density lipoprotein (LDL) levels.

Because our patient presented four primary features (retinal degenerations, polydactyly, obesity, and learning deficits) and six secondary features (behavioral abnormality, delayed development, diabetes mellitus, diabetes insipidus, brachydactyly, and LVH), we diagnosed the case as BBS.

Following proper clinical and laboratory documentations, she was immediately referred to BIRDEM General Hospital's Ophthalmology and Pediatric departments on the same day for further evaluation and management. The patient's mother was encouraged to adhere to the food modification plan and the prescribed oral hypoglycemic medication, thyroxine, lipid-lowering agent, and multivitamin supplements.

There is no cure for this disorder, although early detection and symptomatic, supportive, and rehabilitative interventions can lessen impairment.

### 2.2. Case 2

This 24-year-old male was referred by an endocrinologist with a history of blurry vision in both eyes, especially at night, from 13 years of his age, confirmed by his parents. According to his parents' statement, he had been developing a dimness of vision since childhood, gradually increasing with age. They also noticed poor night vision for the same period, becoming obese, not having normal mental development, and an inability to concentrate on reading or learning. Initially, he was admitted in the primary school at the age of 7 years but could not continue further.

He was the third child of his healthy parents with no history of consanguinity. Unfortunately, his two brothers also had a visual disability without any other physical abnormality, but never went for the proper medical check-up.

**Figure 2:**
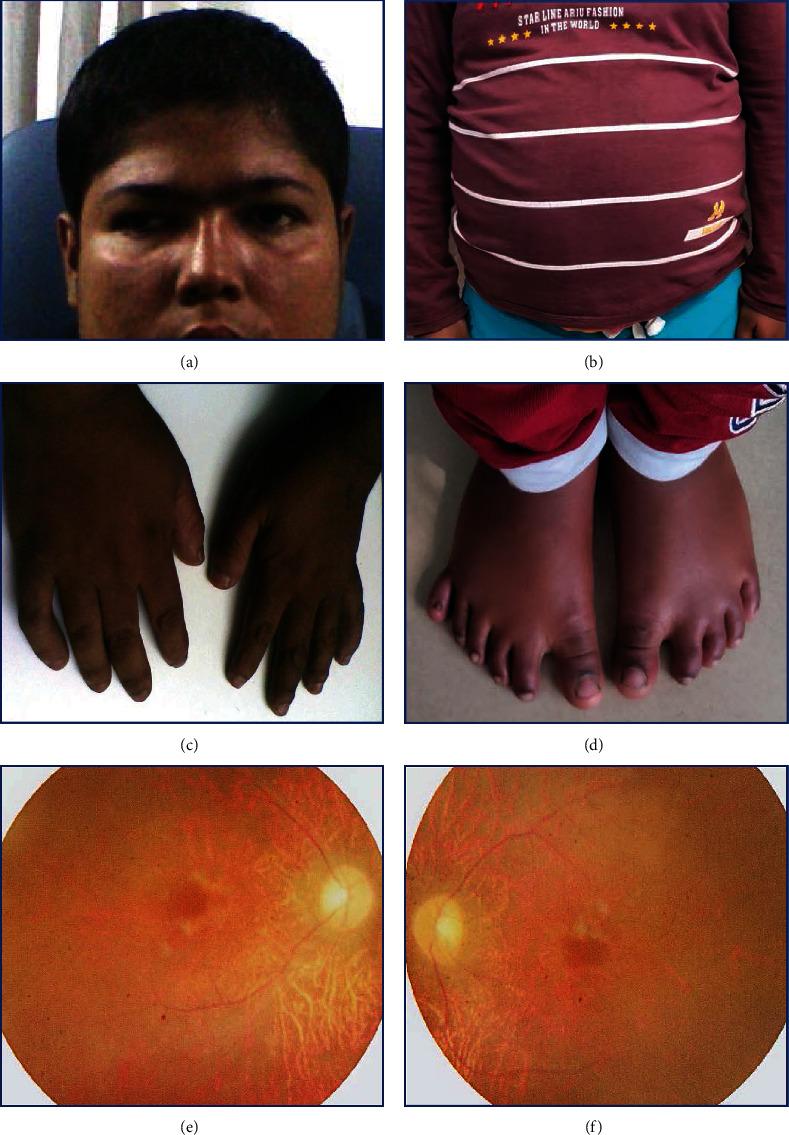
(a) Divergent squint. (b) Central obesity. (c) Short, broad, and stubby fingers of the upper limbs. (d) Polydactyly, syndactyly, and brachydactyly in the lower limbs. (e, f) Fundus images of the right and left eyes showing mild arteriolar attenuation with pigmentary retinopathy and pale optic discs.

While examining the patient, he was restless and irritated, with a weight of 96 kg, a height of 167.64 cm, and a BMI of 34.2 kg/m^2^ (obese) (Figure 2(b)) with below-average intelligence and cognitive impairment in psychiatric evaluation. His vital parameters were within normal limits.

Musculoskeletal system examination revealed brachydactyly in the upper limbs (Figure 2(c)) and polydactyly, syndactyly, and brachydactyly in the lower limbs (Figure 2(d)). Micropenis was also present while examining the urogenital system.

On ophthalmological evaluation, his visual acuity was 6/60 in both eyes with no improvement with pinhole and alternate exotropia of around 45 PD for both distance and near (Figure 2(a)).

Slit-lamp examination showed early lental opacity in both eyes.

Dilated fundus evaluation of both eyes showed pale optic discs, mild arteriolar attenuation, few midperipheral intraretinal perivascular ‘bone spicule' pigmentations, and Bull's eye maculopathy (Figures 2(e) and 2(f)).

Routine investigations, including complete blood count, urine analysis, renal function tests, chest X-ray, and ECG, showed nothing abnormalities. His serum lipid profile revealed markedly elevated cholesterol (258 mg/dL; normal: <200 mg/dL) and TG (253 mg/dL; normal: <150 mg/dL) levels; HDL and LDL were within normal limits.

The hormonal analysis ended up with the result of serum testosterone: 5.8 pg/mL (normal: 35-155 pg/mL; hypogonadism), luteinizing hormone (LH): 3.13 IU/L (normal: 1.8 to 8.6 IU/L in male), free thyroxine (FT4): 11.11 pmol/L (normal: 12 to 30 pmol/L), and thyroid-stimulating hormone (TSH): 0.89 mU/L (normal: 0.4-4.5 mU/L), and impaired oral glucose tolerance test (75 g OGTT) (fasting: 4.2 mmol/L and two-hours after glucose intake: 9.5 mmol/L).

Finally, we diagnosed this as a case of BBS with five major criteria (truncal obesity, polydactyly, retinal dystrophy, learning disabilities, and hypogonadism) and six minor criteria (strabismus and cataract, delay in speech, behavioral disorder, developmental delay, brachydactyly and syndactyly, and impaired glucose tolerance test).

As there is no definitive treatment for this condition, the patient was treated with hormonal and vitamin supplements, lipid-lowering drug with dietary modification according to the pediatrician's advice. We referred the patient to the low-vision clinic for visual rehabilitation and counseled the parents about the prognosis and the importance of regular follow-up.

## 3. Discussion

Bardet–Biedl syndrome (BBS) is a rare autosomal recessive condition with clinical and genetic diversity. There are twenty-one known BBS genes (BBS1-BBS20 and NPHP1). BBS1 and BBS10 are the two primary genes linked with BBS, and these gene alterations were detected in more than 20% of cases [7]. Because genetic analysis is costly and time-consuming, it is reserved for complex situations and experiment purposes. In our cases, genetic testing was not performed due to a lack of availability in our country.

Consanguinity has been a major contributor to the prevalence of this disease. It is widely performed in a number of Middle Eastern nations, including Kuwait, Saudi Arabia, Iran, and Pakistan [8]. Our two cases, however, did not provide a history of the parents' consanguineous marriage.

Table 1 describes the primary and secondary characteristics of BBS. The diagnostic criteria for BBS are either four major characteristics or three primary features and two secondary features. In case 1, four primary and six secondary features were detected, whereas in case 2, five primary and six secondary features were found, fulfilling the BBS diagnostic criteria.

Most features of Bardet–Biedl syndrome (BBS) manifest after many years of development and are not visible during early childhood. The diagnosis of BBS remains challenging in young children due to the gradual development of clinical characteristics. In one major population-based survey conducted in the United Kingdom, the average age of diagnosis was nine years [1]. Case 1 was diagnosed when she was nine, whereas case 2 was diagnosed when he was 24. Given its poor prognosis due to life-threatening symptoms, a strong index of suspicion is necessary for early detection.

One of the most common signs of BBS is central obesity, which generally appears in the first year of life. According to the WHO classifications of body mass index, 72% of post-pubertal participants in a series reported by Beales and Elcioglu were overweight (BMI > 25 kg/m^2^), with 16% having BMI > 40 kg/m^2^, which was categorized as severely obese [1]. Both of our patients had BMIs of 32 kg/m^2^ and 34.2 kg/m^2^, putting them in the obese (BMI > 30 kg/m^2^) group of the WHO classification.

Various forms and frequencies have been found in Limb deformities. The most common abnormalities are polydactyly (63-81%) and brachydactyly (6-100%) of both upper and lower limbs. A significant gap between the first and second toes, fifth finger clinodactyly, and partial syndactyly are all disorder symptoms [1]. Our case 1 had polydactyly in the upper limbs and tiny, stubby fingers in the lower limbs. In contrast, case 2 had brachydactyly in the upper limbs and polydactyly, syndactyly, and brachydactyly in the lower limbs.

One of the essential diagnostic criteria for BBS is learning disability. Low IQ and various visual problems have been encountered for this condition. Lately, only a minority of mentally disabled patients are determined by objective IQ tests. Around 44% of BBS cases have been found with an IQ level of 79 or below. A correlation has been made between the decreased IQ level and visual handicap [1]. Here, cases 1 and 2 had both mental retardation and reduced visual acuity.

Hypogonadism and genital abnormalities are seen in 59-98% of individuals, with men being more affected than females. The short penile length has also been seen in all males with BBS [1]. There was a micropenis in our case 2.

Kidney disease affects 53-82% of patients with BBS, representing the common cause of morbidity and mortality. No morphological and functional renal abnormalities were found in these cases. However, there are various renal abnormalities, such as morphological (parenchymal cysts, dysplastic kidneys, and calyceal clubbing) and functional (chronic renal failure, renal calculi, and vesicoureteric reflux) [1].

Some of the most prevalent fundus abnormalities identified in such individuals include a macular scar with or without an epiretinal membrane, optic disc atrophy (“waxy” disc pallor), perivascular retinal pigmentary changes in “bony spicule” pattern or pigment clumping, and extensive retinal artery constriction. Aside from retinal degeneration, additional ocular symptoms of the condition include refractive errors (mainly myopia), exotropia, and defective color vision. Degenerative changes in the maculae of patients are detected early on, with a persistent loss in their central vision, turning them legally blind by the age of 30 [1]. Our patients had the mentioned characteristic fundus features with bilateral early lental opacity and alternate divergent squint in case 2.

Diabetes mellitus type 2 has been studied in 6-48% of BBS patients; type 1 has also been reported on occasion [9]. Our case 1 had diabetes mellitus, and case 2 had an impaired glucose level; both required dietary changes and physical activity to lose weight.

Valvular stenosis, atrial/ventricular septal abnormalities, or cardiomyopathy is seen in a minority of people and can be identified during pregnancy or after birth. On the other hand, anosmia, hearing loss, liver diseases, Hirschsprung disease, and laterality problems have been described at various ages of development [4]. Our case 1 had LVH characteristics on ECG and electrocardiography.

Patients with BBS are diagnosed using a combination of medical history, family history, examination findings, laboratory investigations, and genetic testing. In terms of medical history, any individual who shows any of the key features listed in Table 1 should be tested for BBS. The core of care is early diagnosis of the condition. In the case of BBS, a comprehensive approach is essential.

Early childhood obesity should be treated through a healthy diet, physical activity, and training. Obesity can be controlled with a nutritious, carbohydrate-reduced diet and frequent cardiovascular activity, such as walking and cycling, with adaptations for blindness. A companion to encourage activity and a good diet might be beneficial. As with our patients, a lack of well-trained dietitians in resource-limited regions, as well as blindness, represent substantial challenges in nutrition management for BBS patients. In the general population, metabolic syndrome and other obesity-related problems of BBS should be treated. Accessory digits can be surgically removed for aesthetic reasons.

Patients with BBS should have regular ophthalmologist consultations. If there is any refractive error, it should be corrected. Patients who are visually handicapped can try low-vision aids. Based on the inevitability of blindness, early educational strategies should be implemented. When it comes to education, it is necessary to employ Braille, mobility training, adapted living skills, and computing abilities (including voice recognition and transcription software), as well as large-print reading materials.

Following a diagnosis, baseline studies such as blood sugar levels, renal function tests, abdominal ultrasound, urine analysis, and refraction are required, and treatment should be initiated accordingly. Finally, genetic counseling can benefit affected individuals and their families. The disease should also be discussed with parents or patients, and they should be followed up regularly, with the frequency depending on the severity of their problem.

## 4. Limitations

A genetic investigation to validate the diagnosis of BBS was not carried out due to its unavailability in Bangladesh.

## 5. Conclusions

In a developing country like Bangladesh, BBS is diagnosed by clinical examination and traditional lab tests. The syndrome's genetic and molecular research will take longer to implement. Because renal impairment is the primary cause of mortality in BBS patients, accurate diagnosis is crucial for preventing the progression of renal impairment. BBS is managed in a supportive manner by a multidisciplinary team, and genetic counseling for families is essential.

## Figures and Tables

**Table 1:** Diagnostic criteria for Bardet–Biedl syndrome (BBS).

	Case 1	Case 2
Primary features		
Truncal obesity (72-92%)	+	+
Polydactyly (63-81%)	+	+
Retinitis pigmentosa/retinal dystrophy (93%)	+	+
Learning disabilities (61%)	+	+
Renal malformations (53%)	—	—
Genital abnormalities (female) (59-98%)	—	—
Hypogonadism (male) (59-98%)	—	+
Secondary features		
Strabismus/cataract/astigmatism	—	+
Speech disorders/delay (54-81%)	+	+
Developmental delay (50-91%)	+	+
Brachydactyly/syndactyly (6-100%)	+	+
Behavioral disorders	+	+
Diabetes mellitus (6-48%)	—	+
Polyuria/polydipsia (diabetes insipidus)	+	—
Left ventricular hypertrophy (LVH)	+	—
Congenital cardiac abnormalities	—	—
Hepatic fibrosis	—	—
Craniofacial dysmorphism (51%)	—	—
Dental crowding/high-arched palate/hypodontia/small roots	—	—
Hirschprung disease	—	—
Ataxia/poor coordination (40-86%)	—	—

## Data Availability

Data sharing is not applicable to this article as no datasets were generated or analyzed during the current study.

## Additional Points

*Disclaimer*. It was performed as a part of academic interest at BIRDEM General Hospital, Dhaka, Bangladesh.

## References

[1] Beales P. L., Elcioglu N., Woolf A. S., Parker D., Flinter F. A. (1999). New criteria for improved diagnosis of Bardet-Biedl syndrome: results of a population survey. *Journal of Medical Genetics*.

[2] Elawad O. A. M. A., Dafallah M. A., Ahmed M. M. M. (2022). Bardet-Biedl syndrome: a case series. *Journal of Medical Case Reports*.

[3] Williams B., Jenkins D., Walls J. (1988). Chronic renal failure; an important feature of the Laurence-Moon-Biedl syndrome. *Postgraduate Medical Journal*.

[4] Forsythe E., Beales P. L. (2013). Bardet-Biedl syndrome. *European Journal of Human Genetics*.

[5] Forsythe E., Kenny J., Bacchelli C., Beales P. L. (2018). Managing Bardet–Biedl Syndrome—Now and in the future. *Frontiers in Pediatrics*.

[6] Bardet G. (1995). On congenital obesity syndrome with polydactyly and retinitis pigmentosa (a contribution to the study of clinical forms of hypophyseal obesity) Thesis, for the degree of Doctor of Medicine. *Obesity Research*.

[7] Mandal R. K., Pande R., Rajani Shah K. C., Acharya B. (2021). Bardet-Biedl syndrome: a case report from Nepal. *Asian Journal of Medical Sciences*.

[8] Maria M., Lamers I. J. C., Schmidts M. (2016). Genetic and clinical characterization of Pakistani families with Bardet-Biedl syndrome extends the genetic and phenotypic spectrum. *Scientific Reports*.

[9] Foggensteiner L., Beales P., Turner N. (2015). *Bardet-Biedl Syndrome and Other Ciliopathies*.

